# Initial Orbit Determination of Low-Earth-Orbiting Objects Using Multi-Site Space-Based Optical Observations

**DOI:** 10.3390/s26206400

**Published:** 2026-10-10

**Authors:** Marianna Veltri, Vithurshan Suthakar, Regina S. K. Lee

**Affiliations:** Department of Earth and Space Science, York University, Toronto, ON M3J 1P3, Canada

**Keywords:** Space Situational Awareness, star-trackers, Initial Orbit Determination, resident space objects, low Earth orbit, optical observations, single-site, multi-site, space-based

## Abstract

Space Situational Awareness (SSA) is a growing field of study which provides knowledge of Resident Space Objects (RSOs) to help maintain a safe and sustainable space environment around Earth. A primary component of SSA is obtaining Initial Orbit Determinations (IODs) of these objects via optical observations. The present way of obtaining optical observations is by using a single observation site. However, using only one observation site does not yield accurate range or IOD results. This research tests space-based single-site and multi-site observations of RSOs in Low Earth Orbit (LEO) to provide the necessary information to calculate IODs. Gooding’s algorithm is used to calculate IODs from the simulated data, since it is the most robust IOD algorithm discussed in the literature. The results from this research demonstrate that selected multi-site observations produce more accurate range and IOD results than single-site observations and, thus, provide advancement opportunities for various SSA efforts.

## 1. Introduction

**Figure 1:**
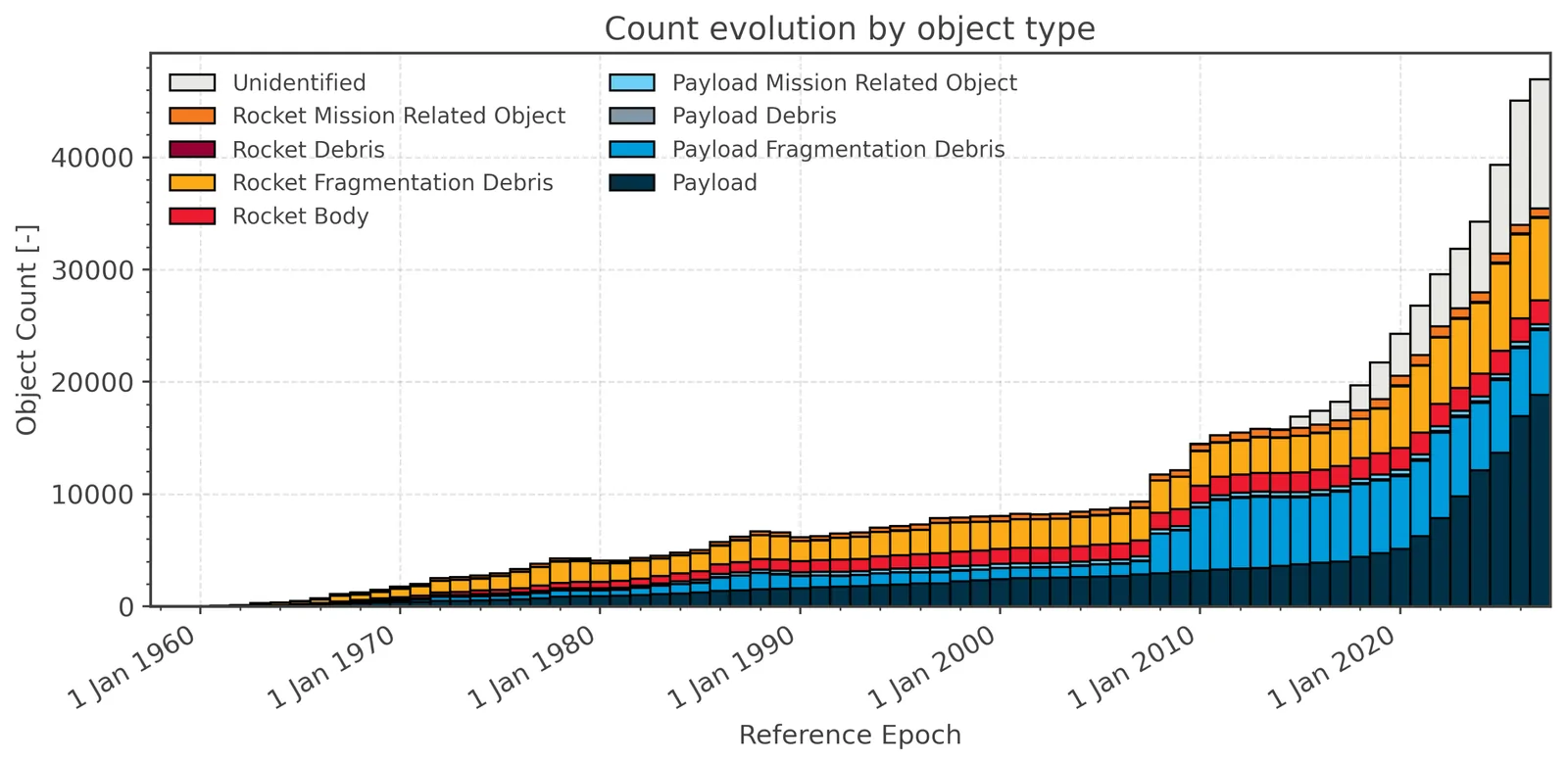
The count evolution for the total number of RSOs that orbit Earth. The RSOs are grouped by object type, some of which include fragmentation debris and unidentified objects. Image Credit: [1].

There are over 140 million Resident Space Objects (RSOs) in orbit around Earth, and currently only around 44,000 of these objects are regularly tracked and maintained in a catalog [1]. A large majority of RSOs reside in Low Earth Orbit (LEO); however, they can also be found in all other orbits, such as Medium Earth Orbit (MEO) and Geostationary Orbit (GEO). With the vast majority of these objects unaccounted for, the risk of unpredictable collisions is high. Furthermore, the RSO population is growing at a rapid pace due to continuous new launches, fragmentation, and collisions [2]. The evolution of the number of objects in orbit around Earth over many years can be seen in Figure 1 [1]. Due to this increasing RSO congestion, there has been a push for development in RSO orbital research.

Space Situational Awareness (SSA) is a growing field of study that provides knowledge of RSOs and their orbits to help maintain a safe and sustainable space environment around our planet. The primary objective of SSA is to obtain the orbital information of RSOs and to increase the accuracy of their known orbits. By doing so, collision prediction and prevention methods are possible. With constant new launches and frequent fragmentation events, the number of RSOs is steadily rising, thus further increasing the demand for precise orbital information. A large component of SSA is obtaining these orbital characteristics through Initial Orbit Determination (IOD). Presently, IOD is commonly obtained by using single-site observations; however, this process cannot always provide direct range measurements or extremely accurate IODs [3]. This research instead proposes the use of multi-site observations to obtain more accurate range and IOD measurements.

**Figure 2:**
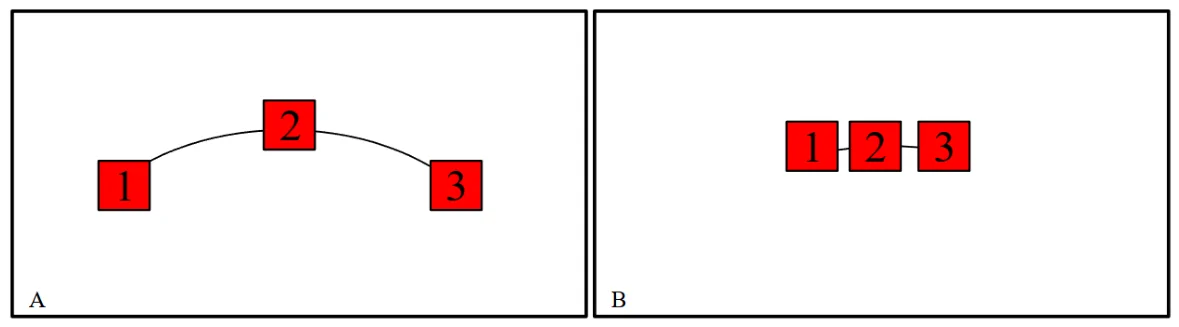
Examples of observational arcs. The red boxes represent the RSO being observed. The red boxes with the number ‘1’ represent the first observations. The red boxes with the number ‘2’ represent the second observations. The red boxes with the number ‘3’ represent the third observations. Image (**A**) represents an arc of the RSO’s orbit, which is formed from the three observations. Image (**B**) represents the too-short-arc problem.

IOD is the first step in orbital research, which does not require any prior information on an RSO. IOD produces estimates of the RSO’s orbit based on the minimum amount of observations of the object. This preliminary orbit estimate only requires three angles-only observations of an RSO and is the foundation for obtaining an accurate orbital understanding of these objects. IOD must be accurate, as it creates a starting point for collision prediction and prevention methods for SSA endeavors. Angles-only observations are performed using optical imagers, where the ascension and declination of an RSO can be obtained from an image and used to calculate its position vector [4]. IOD calculations require an RSO to be observed at three separate instances to form an arc of its full orbit and to determine the RSO state vectors [4]. An example of this arc can be seen in Figure 2A.

However, there is a common issue in IOD research: the too-short-arc problem. This issue arises when the three observations form an arc that is too small for IOD calculations, due to the limited data used for IOD calculations [5]. An example of this too-short-arc problem can be seen in Figure 2B. Furthermore, a too-short-arc problem can arise for a variety of reasons, one of which is the distance between the host(s) and the RSO. When the host(s) are located in LEO, observations of RSOs in MEO or GEO may be more susceptible to forming too-short arcs due to the large distance between the host(s) and RSO. As this distance increases, the possibility of encountering a too-short-arc problem also increases, particularly when the observation time span remains short. Consequently, simultaneous multi-site observations of RSOs in MEO and GEO, from LEO hosts, may not provide an advantage for IOD. The increased distance between the hosts and RSO, along with the simultaneous observations, could produce observations that look more like point sources rather than arcs, which can impose large restraints on the ability of IOD methods to accurately determine the RSO’s orbit. One approach to mitigate the effects of a too-short-arc problem arising in these scenarios is to increase the observation time span. Longer observation periods provide a larger observation arc of the RSO, which reduces the likelihood that the observations will form an insufficient short arc. The specifics of this, however, are based on a case-by-case scenario.

Similarly, IOD is the initial step in orbit determination (OD), where preliminary orbital estimates are refined. As discussed in [6], numerous OD methods exist, with varying levels of accuracy, making the quality of IOD estimates a critical factor in the overall OD process. The importance of accurate IOD and OD is well documented throughout the literature [6]. Furthermore, the increasing number of objects in orbit around Earth and, thus, the increasing risk of collisions have highlighted the need for highly accurate orbit determination techniques [6].

The traditional way to obtain angles-only observations for IOD calculations is by using a single optical observation site. This one observation site can either be ground-based or space-based. Since IOD requires observing an RSO at three instances, these observations are obtained from the same (one) observer, usually at different time periods. The too-short-arc problem commonly arises in single-site observations because the data is limited and tends to have high degrees of similarity due to originating from the same observer [5]. Because of this, using only one observation site does not always yield accurate range or IOD results, which is a major issue in SSA collision prediction and prevention methods [3].

This research examines the use of multiple observation sites to yield more precise and accurate range and IOD results than those from single-site observations. Since IOD requires observing an RSO at three instances, these observations are obtained from different (multiple) observers. Having multiple observers provides the ability to obtain simultaneous observations of an RSO and decreases the possibility of having a too-short arc for IOD calculations. Simultaneous observations also provide direct range estimates, which are a crucial part of IOD calculations. The use of multi-site observations has been a topic in future work sections within IOD, so this research will be a strong foundation for new IOD studies. Some possible advantages for multi-site observations include the following:

1. Observational Arcs: With multiple observers, data can be taken over a longer period of time, thus decreasing the possibility of having too short an arc for IOD calculations.
2. Simultaneous Observations: Multiple observers can obtain observations of the same RSO simultaneously, allowing for more straightforward range calculations
3. Improved Orbital Accuracy: Having more accurate range estimates as inputs for IOD algorithms allows for more accurate IOD calculations

Building from these advantages, this research has four main objectives, which are either touched upon in the rest of this study or help shape the data collection process and the analysis of the results:

1. To assess the feasibility of star trackers as optical sensors for angles-only IOD of RSOs.
2. To evaluate whether multi-site space-based observations can yield more accurate IOD results than single-site observations by systematically comparing single-host, double-host, and triple-host configurations.
3. To investigate whether host-satellite constellation orientation—specifically, a straight-line versus a diagonal-line formation—has an effect on IOD accuracy
4. To test IOD sensitivity regarding angular noise between the single-site scenario and the overall best multi-site scenario.

Optical observations provide angles-only data on the RSO at three given times, which is necessary to calculate the RSO’s state vectors [4]. These observations can be done via ground-based or space-based observations, each with its own advantages and drawbacks. Ground-based sites typically have larger apertures than space-based sites and are more sensitive [7]. Furthermore, there are many ground-based sites that are open and accessible for research purposes worldwide. The global distribution of these ground-based sites is useful for obtaining large sky coverage to maximize the number of RSO observations [7]. Along with this, maintenance and real-time solutions for issues are possible, since the instruments are easily accessible. However, ground-based observations are greatly affected by weather and atmospheric conditions and are usually only useful for nighttime observations. In multi-site observations, the regular weather and atmospheric difficulties must be considered for all locations, which likely have different conditions. Similarly, for simultaneous multi-site ground-based observations, the instrumentation at all sites must be identical, and the field of view (FOV) of these instruments must line up. Additionally, research using multiple ground-based observers revealed that the distance between the ground observers highly affects the accuracy of the IOD [8]. Multiple ground-based sites can lead to more unnecessary variables affecting IOD accuracy, since the locations of pre-established ground observation locations cannot be modified.

Conversely, space-based observations offer advantages such as immunity to atmospheric and weather conditions and are minimally affected by daylight. Space-based observations also have the ability to observe with wider angles and better flexibility, which can lead to larger amounts of data being collected in comparison to ground-based observations [6]. A single space-based observer has the ability to make multiple revolutions around its orbit and obtain numerous observations of the same RSO. Having multiple space-based observers increases the number of instances in which the same RSO can be observed, maximizing the amount of data collected. Additionally, multi-site observations allow for direct range calculations between the hosts and the RSO. One approach for SSA space-based observations is re-purposing existing sensors on satellites such as star trackers. These star trackers provide a cheap way to obtain observations of RSOs with all of the needed information to further obtain IODs [9,10]. Thus, this research uses space-based observations stemming from a star-tracker-like imager, hence avoiding the complexities that are brought forth from atmospheric interference experienced by ground-based systems.

A prominent space-based observation technique being discussed in the literature is the use of star trackers for RSO observations. Star trackers, whose main purpose is for attitude determination, have proven to be useful in detecting and observing RSOs, as seen in [11,12,13,14]. The validity of using star trackers for RSO observations was tested and confirmed within [11,15,16], where the results prove that star trackers are a promising technology that could support future RSO IOD research. Since IOD from optical observations requires angles-only calculations, refs. [9,17] discuss in detail how one can obtain the angles-only data from star-tracker images, further proving their effectiveness. Furthermore, refs. [13,14] tested the possibility of using multiple star trackers to observe the same RSO. Since there are many star trackers in orbit, ref. [13] used simulations to confirm that it is possible to observe the same RSO from two different star trackers and calculate their orbits. The work within [13] simulated three space-based star trackers with target altitudes from approximately 6800 to 40,000 km and observation distances of 50–200 km. The resulting orbit determination errors were below 100 m for low-altitude targets and below 1000 m for high-altitude targets [13]. Along with this, ref. [14] confirmed that multiple star trackers could detect RSOs. The work in [14] investigated two-site optical observations for GEO satellites using ground-based observations with a baseline of approximately 1000 km. They reported comparisons of orbit determination accuracy from a variety of methods while defining TLE data as the truth data [14].

There is a large gap within IOD research, since extremely minimal research has been done on multi-site observations with respect to IOD. Refs. [13,14] are the only relevant literature in this area of study; however, they only cover an extremely small portion of the missing research. The objectives of both [13,14] differ from those of this research, where our main objectives involve comparing space-based single-, double-, and triple-host observations, testing near-simultaneous observations, and analyzing the effects of multi-site constellation orientations. The objectives from our work build a stronger foundation for space-based multi-site observations, specifically using star trackers, within IOD research. Our work, along with the evidence that star trackers could be used for RSO observations, is ideal, since it allows for single-site and multi-site observations of RSOs without needing to launch new satellites into orbit and add to the congestion of RSOs.

Many different IOD methods use angle-only observations and calculate the respective orbits. Some of these methods include Gauss’s method, Laplace’s method, and Gooding’s method, which are commonly found within the literature [18,19,20]. Laplace’s method requires angles-only observations of the RSO and the position data of the host to determine the RSO’s velocity and acceleration [18]. Additionally, his method was first developed for the orbit prediction of natural celestial objects and not artificial satellites; thus, it is not ideal for RSO IOD calculations [4,18]. Similarly, Gauss’s method requires angles-only observations of the RSO to determine its position and velocity vector [19]. Gauss’s method is most accurate when used for interplanetary studies; however, it is useful for RSO IOD calculations under certain scenarios [4,19]. Although those two IOD methods are some of the most common within orbit research, Gooding’s method is far more robust for RSO IOD calculations [20]. While an in-depth description of Gooding’s algorithm can be found within his report, his algorithm essentially uses a high-order Newton correction and solves Lambert’s orbital boundary-value problem [20]. Gooding’s algorithm iteratively refines range estimates and has no limitations on the three observations’ locations, making it ideal for multi-site scenarios [20].

There has been extensive study on the various IOD algorithms to determine which is the most robust and produces the most accurate results. Using simulated data, ref. [21] tested four different IOD algorithms for RSOs located in LEO: Laplace, Gauss, Double-r, and Gooding’s method. The results for the various scenarios demonstrate that Gooding’s algorithm is the most robust for many of the scenarios [21]. Similarly, ref. [22] tested Laplace’s, Gauss’s, Baker-Jacoby’s, and Gooding’s methods on objects from a variety of different orbits. These results further demonstrate that Gooding’s algorithm performs the best compared to all of the other methods for a large majority of the scenarios [22]. It is also important to note that Gooding’s algorithm has no restrictions on the three observing sites, thus making it suitable for various scenario testing of multi-site observations [20]. From this, ref. [13] is successful in using Gooding’s algorithm for calculating the IOD of the RSOs. It is due to the continuous evidence found in [4,13,21,22], as well as the fact that Gooding’s algorithm has no restrictions on the three observation sites, that Gooding’s algorithm was chosen as the IOD algorithm for this research [20].

Based on this body of literature, Gooding’s algorithm was selected for this study. Although prior studies have demonstrated the feasibility of using star trackers for RSO detection and have shown that Gooding’s method is effective for angles-only IOD, there remains limited systematic analysis of how observer multiplicity, observation timing, and formation geometry affect orbit-determination accuracy in space-based multi-site scenarios. This study addresses that gap by comparing single-site, two-site, and three-site observations of LEO RSOs under normal and near-simultaneous timing conditions and under two host-satellite formation geometries.

This research supports the prospect of using star trackers not only for RSO detection but also for IOD calculations. The data for this research was collected through simulations using the Ansys System Tool Kit and is described in detail in Section 2 [23]. This study focuses on RSOs in LEO, which represents the most densely populated and operationally critical orbital regime [1]. Similarly, the host satellite platforms are likewise placed in LEO and are modeled after the Cascade Smallsat and Ionospheric Polar Explorer (CASSIOPE) satellite [23,24,25]. The primary objective is to assess whether multi-site space-based observations yield more accurate IOD results than single-site observations, evaluated across single-host, dual-host, and triple-host configurations. Two additional variables are examined: the effect of inter-observation timing, comparing near-simultaneous observations (≤40 s separation) against temporally spaced observations (≥300 s separation); and the effect of host-satellite constellation geometry, comparing a collinear (straight-line) formation against an offset (diagonal-line) formation. Nine distinct scenarios are evaluated in total, with the results presented and analyzed in Section 3. The full set of access reports from which these inputs are derived, covering all 10 RSOs across all nine simulation scenarios, is provided in the accompanying dataset [26].

## 2. Methods

### 2.1. Simulation Methodology and Experimental Design

Publicly available multi-site space-based observations of RSOs do not currently exist, so this research uses simulated scenarios constructed from real ephemeris and TLE data within the Ansys Systems Tool Kit (STK) version 13 (Ansys, Canonsburg, PA, USA) [23]. All scenarios tested are idealized observation geometries used to investigate geometric effects; however, formation-flying dynamics and station-keeping are outside the scope of the present study. All hosts are modeled after CASSIOPE (University of Calgary, Calgary, AB, Canada), and with ephemeris sourced from [24] and sensor parameter specifications from [25]. Hosts are simulated using real CASSIOPE ephemeris data, which spans an entire day and is in the geocentric equatorial inertial frame, also known as the Earth-Centered Inertial (ECI) frame [24]. All coordinate information on CASSIOPE (University of Calgary, Calgary, AB, Canada) can be found at [27] and is kept constant through all simulations. The onboard sensor is represented by a conical field of view (FOV) of 26°, consistent with the specifications of the Fast Auroral Imager (FAI) star tracker [25]. Ten RSOs in LEO are created using TLEs and are propagated over time using the SGP4 propagator [23]. This sample size of RSOs is consistent with the values reported in the recent literature, where typically fewer than 10 RSOs were evaluated [8,13,14,28]. All RSO TLE data are represented in the ECI coordinate frame to maintain consistency with the coordinate frame used for the host satellites. The ECI frame is used throughout the simulations and data collection because both the real host and RSO data are provided in this frame. Additionally, Gooding’s algorithm requires all of the input data to be expressed in the ECI frame for IOD calculations.

Nine different scenarios were evaluated, each designed to address one or more of the research objectives outlined in Section 1. The variations between scenarios are described in detail in the following subsections. For all scenarios, the RSO set, observation dates, and observation times are held constant to ensure consistent and directly comparable results. The estimated position, velocity, and range variables from each scenario are evaluated against STK truth data to determine which configuration produces the most accurate IOD results. It is important to note that this research—and, thus, its simulations—focuses only on the IOD problem and does not address the separate data association problem that arises in multi-target observation environments. The observations in these simulations are assumed to have been correctly associated with the corresponding RSO prior to processing, meaning that each IOD calculation belongs to a single known RSO with no ambiguity between the observations. Data association failure can occur in multi-target scenarios; however, the assessment of association methods and their impact on IOD calculations is beyond the scope of this work. The access report data generated from all nine scenarios and used in the IOD analysis are expressed in the ECI coordinate frame and are publicly available [26].

### 2.2. Ansys System Tool Kit (STK)

**Figure 3:**
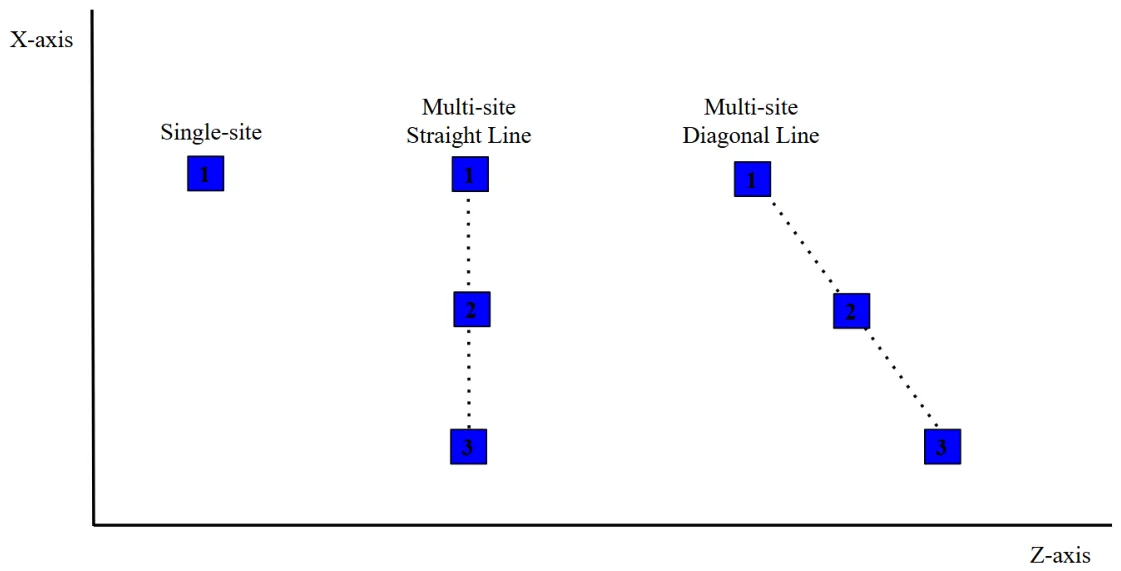
All three orientations for host satellites that are tested in this research: single-site, multi-site straight line, and multi-site diagonal line. Any blue box with the number ‘1’ represents the first host. Any blue box with the number ‘2’ represents the second host. Any blue box with the number ‘3’ represents the third host.

The Ansys Systems Tool Kit (STK) version 13 (Ansys, Canonsburg, PA, USA) is used as a tool to create all single- and multi-site observation scenarios and to generate the input data required for IOD calculations across the 10 LEO RSOs [23]. The STK additionally serves as the source of truth state vectors for all RSOs, against which the IOD outputs are evaluated for accuracy [23]. Orientations for both single- and multi-site observations were configured using CASSIOPE’s (University of Calgary, Calgary, AB, Canada) ephemeris data, which uses the geocentric equatorial inertial frame [24]. The three main orientations tested within this research are single-site, multi-site straight line, and multi-site diagonal line, as shown in Figure 3 and explained throughout this section. For all scenarios, access reports are obtained for each RSO. Access reports contain information on when the RSO is in the FOV of the host’s sensor and provides the information needed to calculate IOD, such as host position vectors and time intervals [23]. The nine scenarios evaluated in this study are as follows:

1. Single-Site: Observations done ≥ 300 s;
2. Normal Two-Site Straight Line: Observations done ≥ 300 s;
3. Simultaneous Two-Site Straight Line: Observations done ≤ 40 s;
4. Normal Three-Site Straight Line: Observations done ≥ 300 s;
5. Simultaneous Three-Site Straight Line: Observations done ≤ 40 s;
6. Normal Two-Site Diagonal Line: Observations done ≥ 300s;
7. Simultaneous Two-Site Diagonal Line: Observations done ≤ 40 s;
8. Normal Three-Site Diagonal Line: Observations done ≥ 300 s;
9. Simultaneous Three-Site Diagonal Line: Observations done ≤ 40 s.

For all multi-site scenarios, observations are collected under two timing conditions. In the ‘normal’condition, the three observations required by Gooding’s algorithm are separated by ≥300 s, ensuring that the RSO traverses a sufficiently large orbital arc to avoid the too-short-arc problem. In the ‘simultaneous’ condition, observations are separated by ≤40 s. The lower bound is not strictly zero due to simulation constraints and the practical difficulty of achieving perfect temporal synchronization across multiple hosts [23]; however, this is consistent with real operational scenarios, in which truly simultaneous multi-site observations are rarely achievable due to geometric and technological limitations. Detailed descriptions of each scenario are provided in the following subsections.

#### 2.2.1. Single-Site Scenario

**Figure 4:**
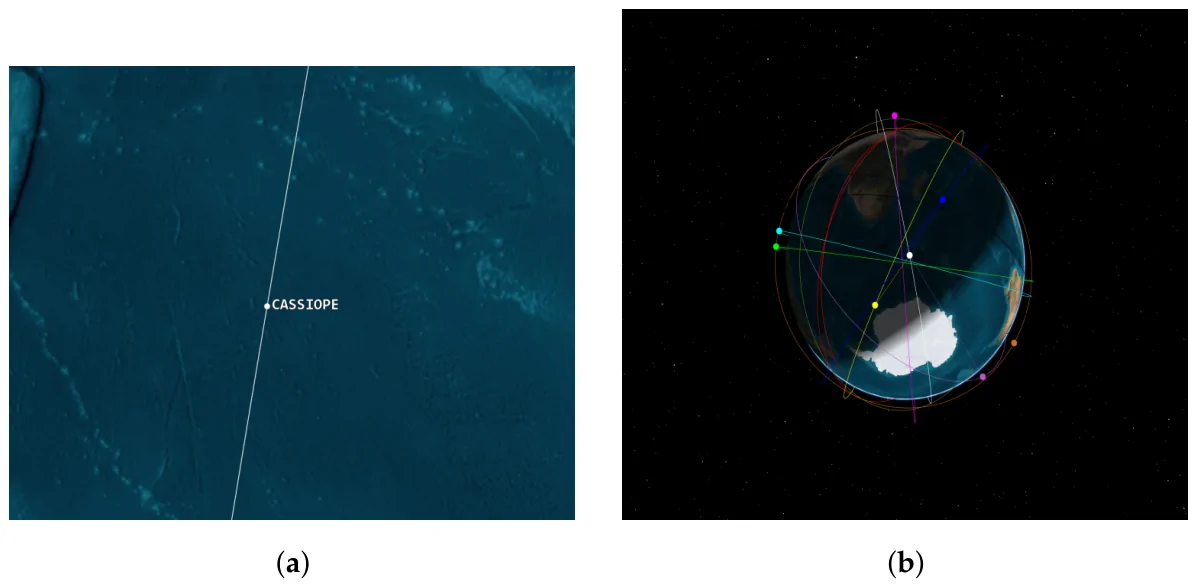
STK simulation of the single-site host and the corresponding scenario containing the single-site host and all 10 LEO RSOs: (**a**) ‘CASSIOPE’ is the single-site host, with the host motion from the bottom of the image to the top of the image; (**b**) the single-site host and all 10 LEO RSOs [23,24,25].

Single-site observations only contain one host, which is created using the original ephemeris data, with no added orbital changes. By using ephemeris and TLE data, both the host and RSO states are grounded in real observational data, providing a physically representative simulation environment. The single-site scenario geometry is illustrated in Figure 4.

#### 2.2.2. Multi-Site Scenarios

The multi-site configurations involve using two and three host satellites. This study limits the multi-site configurations to a maximum of three host satellites to match the three-observation input structure of Gooding’s algorithm. Additional observers beyond three could provide value in practice through overdetermined least-squares orbit refinement, improved numerical conditioning, and outlier rejection; however, such extended formulations are beyond the scope of this initial comparative study. It is important to note again that the multi-site scenarios tested here are idealized observation geometries used to investigate geometric effects, and that formation-flying dynamics and station-keeping are outside the scope of the present study.

The multi-site scenarios are oriented in two ways: in a straight-line or a diagonal-line constellation formation. These orientations were chosen because they form a simple yet physically distinct baseline for formation-flying IOD research, providing an initial reference for future mission design studies. Baselines between ground-based multi-site observers play an important part in the IOD accuracy, as seen in [14], so this research compares the results between the two multi-site scenarios to see if, or how, a space-based baseline affects the overall IOD accuracy. Range values are needed for Gooding’s algorithm, as discussed in Section 2.5, and the calculation of range is highly dependent on the geometry of the host. Hence, if the range estimate has poor accuracy due to the geometry of the host, it will reflect on the overall IOD accuracy and help determine the influence that host geometry has on IOD calculations. This comparison will be particularly interesting regarding the (collinear) straight-line formation, since collinear observer baselines can produce weak IOD due to minimal line-of-sight rotation.

### 2.3. Straight-Line Scenario

**Figure 5:**
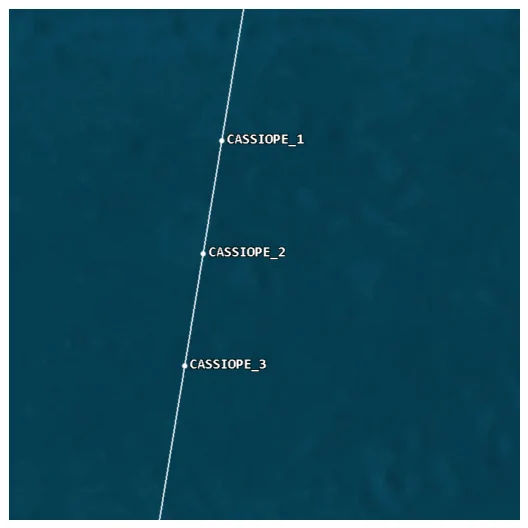
STK simulation of the straight-line multi-site configuration with two and three host satellites, with all host trajectories proceeding from the top to the bottom of the image. ‘CASSIOPE_1’ represents the first host. ‘CASSIOPE_2’ represents the second host. ‘CASSIOPE_3’ represents the third host [23,24,25].

The first host satellite in the straight-line configuration is created identically to the single-site scenario described in Section 2.2.1 [24,25]. The second and third host satellites are derived from the same ephemeris, with their state vectors propagated forward in time by 10 s and 20 s, respectively, relative to the first host. At CASSIOPE’s mean orbital speed of approximately 7.5 km/s, the 10 s and 20 s time offsets correspond to along-track separations of approximately 75 km and 150 km, placing all three hosts in a collinear along-track formation. The baseline length between the first and second hosts is approximately 38.442 km, while the baseline length between the first and third hosts is approximately 84.558 km. Furthermore, the angle between the baselines is approximately 0.18553 degrees. The straight-line scenario geometry is shown in Figure 5.

### 2.4. Diagonal-Line Scenario

**Figure 6:**
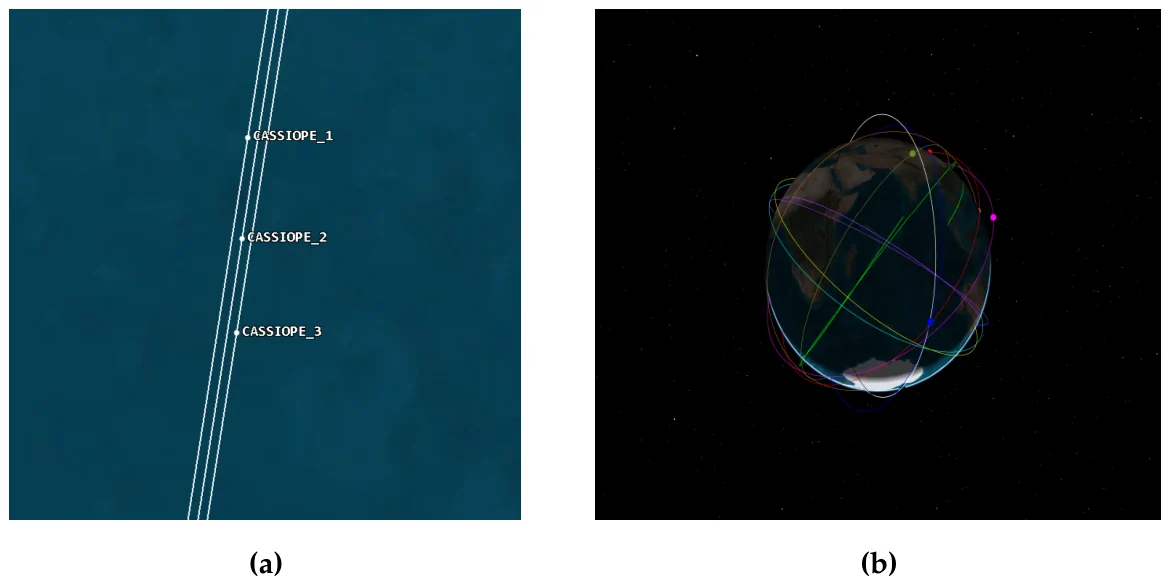
STK simulations of the multi-site diagonal-line configuration and the combined multi-site orbital geometry: (**a**) The diagonal-line formation with two and three host satellites, with all host trajectories proceeding from the top to the bottom of the image. ‘CASSIOPE_1’ represents the first host. ‘CASSIOPE_2’ represents the second host. ‘CASSIOPE_3’ represents the third host. (**b**) All of the multi-site host satellites for both the straight-line and diagonal-line configurations, alongside all 10 simulated LEO RSOs [23,24,25].

Again, the first host in the diagonal-line configuration is created identically to the single-site host described in Section 2.2.1 [24,25]. The second and third host satellites are derived from the same ephemeris and adjusted accordingly [24,25]. The second host satellite is propagated by 10 km in the X axis and by 5 km in the Z axis, while the third satellite is propagated by 20 km in the X axis and by 10 km in the Z axis. The baseline length between the first and second hosts is approximately 7.8856 km, while the baseline length between the first and third hosts is approximately 11.651 km. Furthermore, the angle between the baselines is approximately 16.623 degrees. These offsets produce a diagonal-line formation across all three hosts, as illustrated in Figure 6.

### 2.5. Gooding’s Algorithm

Gooding’s algorithm requires a variety of input data, such as time intervals, host position vectors, line-of-sight (LOS) unit vectors of the RSO, and range estimates [20], where LOS unit vectors are derived from the angles-only data of the RSO with respect to the host observer, while the range is the scalar distance between the host observer and the RSO. It is important to note that Gooding’s algorithm requires the input data to be expressed in the ECI coordinate frame. The algorithm (accessed on 9 April 2026) implementation used in this study was obtained from [29].

IOD calculations require three temporally distinct observations of the RSO. For single-site scenarios, those observations come from a singular host; however, for multi-site scenarios, those observations come from different hosts. The required input values are either extracted directly from the access reports or calculated from the reports’ data [23]. An access report displays data for each time interval during which the host satellite(s) have observational access to the RSO [23]. It consists of numerous lines of data that span the entire time range [23].

In real observational scenarios, range estimates would be obtained through noisy parallax triangulation from the star-tracker images. In our study, since it is simulation-based, the range is computed directly from the host and the RSO truth position vectors provided by the access reports using vector subtraction. Since access reports are very large, Table 1, Table 2, Table 3, Table 4 and Table 5 display examples of lines of data for RSO 15773 that are actually used in the IOD analysis.

Table 1 displays access report data on RSO 15773 from single-site observations, where all three observations are taken from the same host satellite. Table 2 and Table 3 display data for straight-line scenarios, while Table 4 and Table 5 display data for diagonal-line scenarios for example purposes.

The timing of each observation and the position of the host satellite(s) can be taken directly from the access reports (as seen in Table 1, Table 2, Table 3, Table 4 and Table 5). These values are used as direct inputs for Gooding’s algorithm. The position vectors of the host(s) and the RSO are then used to calculate the line-of-sight unit vectors and the range estimates between the objects. This can be achieved through simple vector mathematics. From here, all major input values for Gooding’s algorithm are obtained: time, host position vector, RSO line-of-sight unit vectors, and range estimates. An example of input values for the single-site observations of RSO 15773 and Gooding’s output values can be seen in Table 6 and Table 7.

## 3. Results

The results for all nine observation scenarios are displayed in Table 8, Table 9 and Table 10. These tables display the bias, standard deviation, and root-mean-square error (RMSE) of the results using Gooding’s algorithm and truth data from the STK [20,23,24,25,29]. Table 11 displays the Root Sum Square (RSS) errors of the position values and the RSS errors of the velocity values, for all nine scenarios, to demonstrate a more straightforward view of the results. Green cells mean that that particular scenario has the best result within that column. Rows that are gray mean that the specific scenario cannot be represented by the data properly, meaning that Gooding’s algorithm was not able to process the data for some RSOs for that scenario. This is further discussed in the following sections.

From Table 10, it is evident that the ‘simultaneous’ observations from three host satellites in a diagonal-line formation produce the most accurate results by far for all variables compared to all other scenarios. Most importantly, these results far surpass the results from single-site observations. To support this claim, Table 8 displays the bias for the results, where ‘simultaneous’ observations from three host satellites in a diagonal-line formation performed better than single-site observations as a whole. While the ‘normal’ observations from two host satellites in a diagonal-line formation did perform slightly better for the X position of the RSO, the ‘simultaneous’ observations from three host satellites in a diagonal-line formation were not far off.

Similarly, Table 9 displays the standard deviation for the results, and again, the ‘simultaneous’ observations from three host satellites in a diagonal-line formation performed better than single-site observations as a whole. While the ‘simultaneous’ observations from two host satellites in a straight-line formation produced better results for range estimations, the ‘simultaneous’ observations from three host satellites in a diagonal-line formation were a very close second. It is important to note that Gooding’s algorithm needs range estimates from the first and third observations as inputs, so that it can iteratively refine these range estimates [20]. This algorithm also uses data from the second observation time for the IOD calculations, which are identical in these scenarios, thus producing more accurate results. It is believed that the ‘simultaneous’ observations from two host satellites in a straight-line formation obtained slightly better standard deviations for the range estimates because the middle observation data and the third observation data were exactly the same, since they were from the exact same host and time, as shown in Table 2.

Furthermore, Table 11 displays the RSS errors of the position values and the RSS errors of the velocity values for all nine scenarios. The results in this table further emphasize the significant decrease in error from the simultaneous three-site diagonal-line scenario. This straightforward view of the results is consistent with the discussion and the conclusions drawn in Section 3 and Section 4.

**Figure 7:**
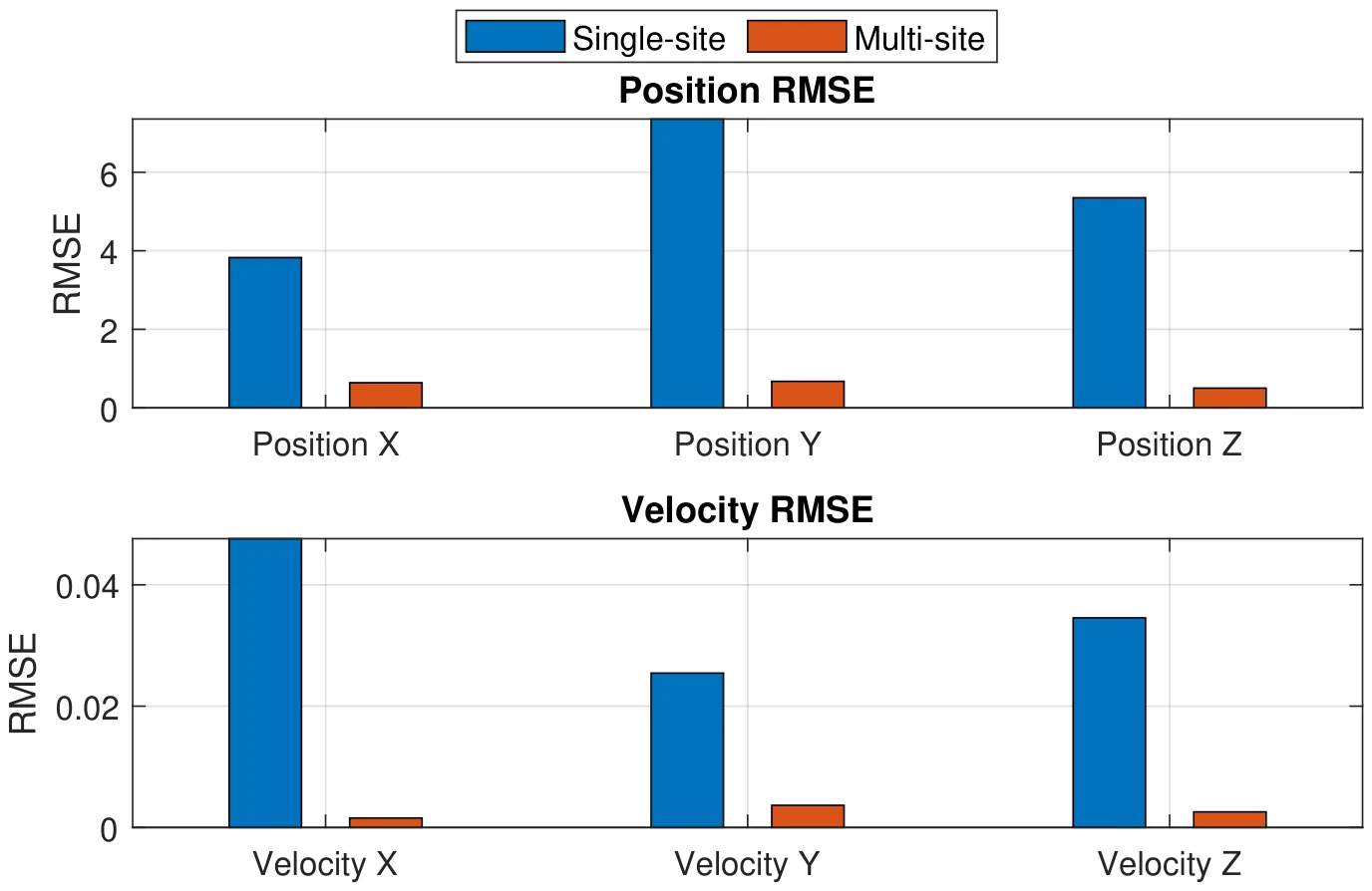

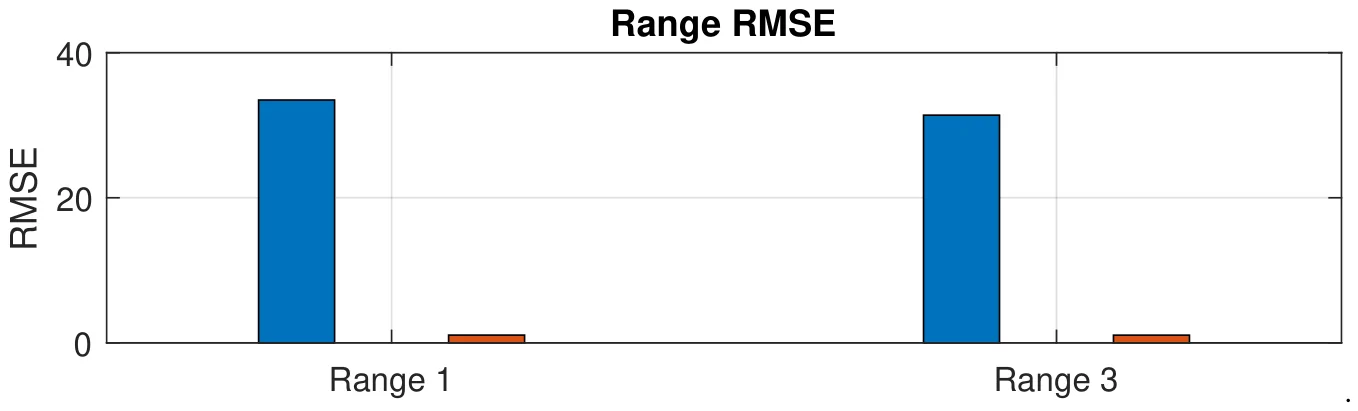
The RMSE comparison between the single-site scenario (blue) and the ‘simultaneous’ observations from three host satellites in a diagonal-line formation (orange).

**Figure 8:**
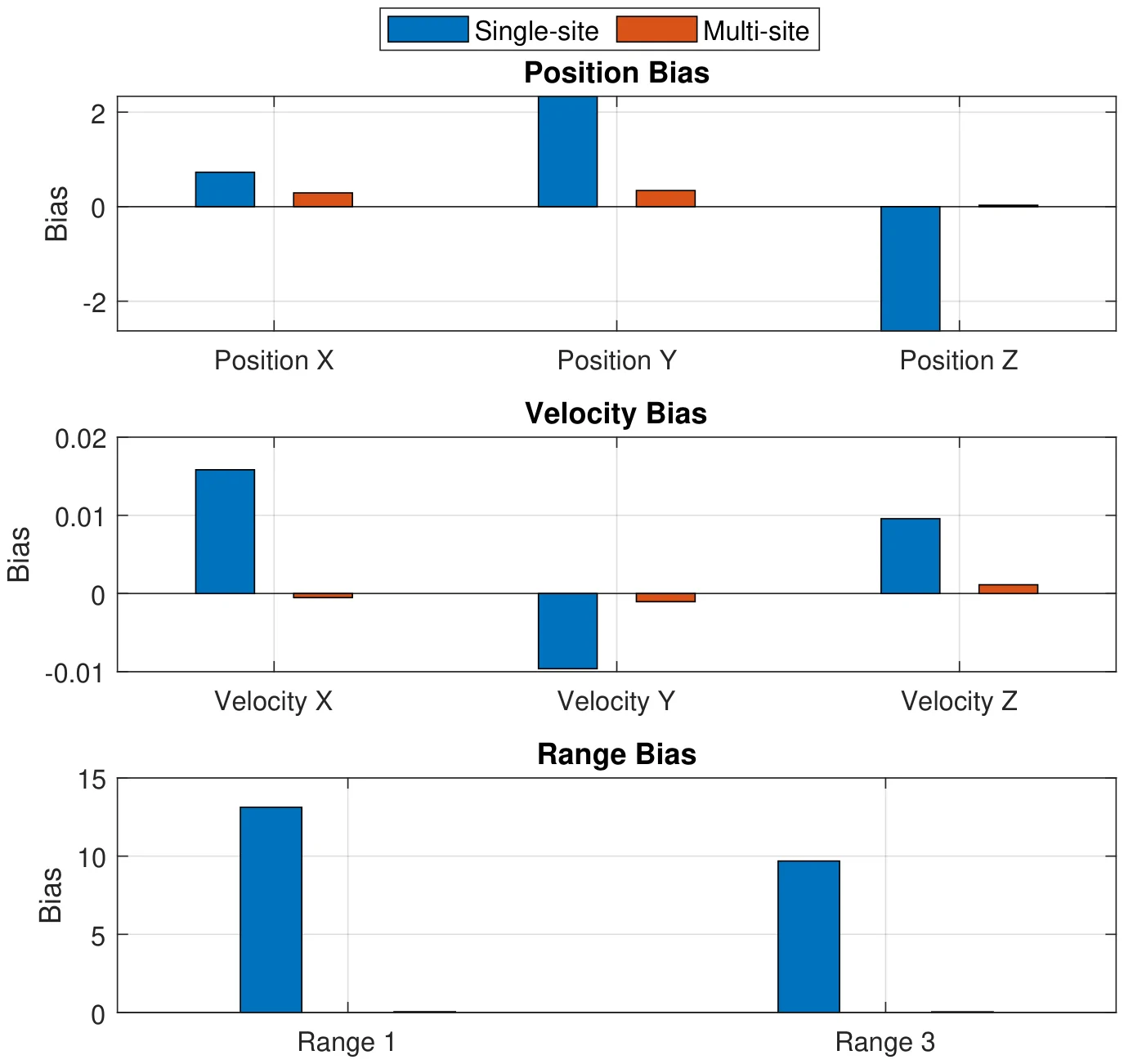
The bias comparison between the single-site scenario (blue) and the ‘simultaneous’ observations from three host satellites in a diagonal-line formation (orange).

**Figure 9:**
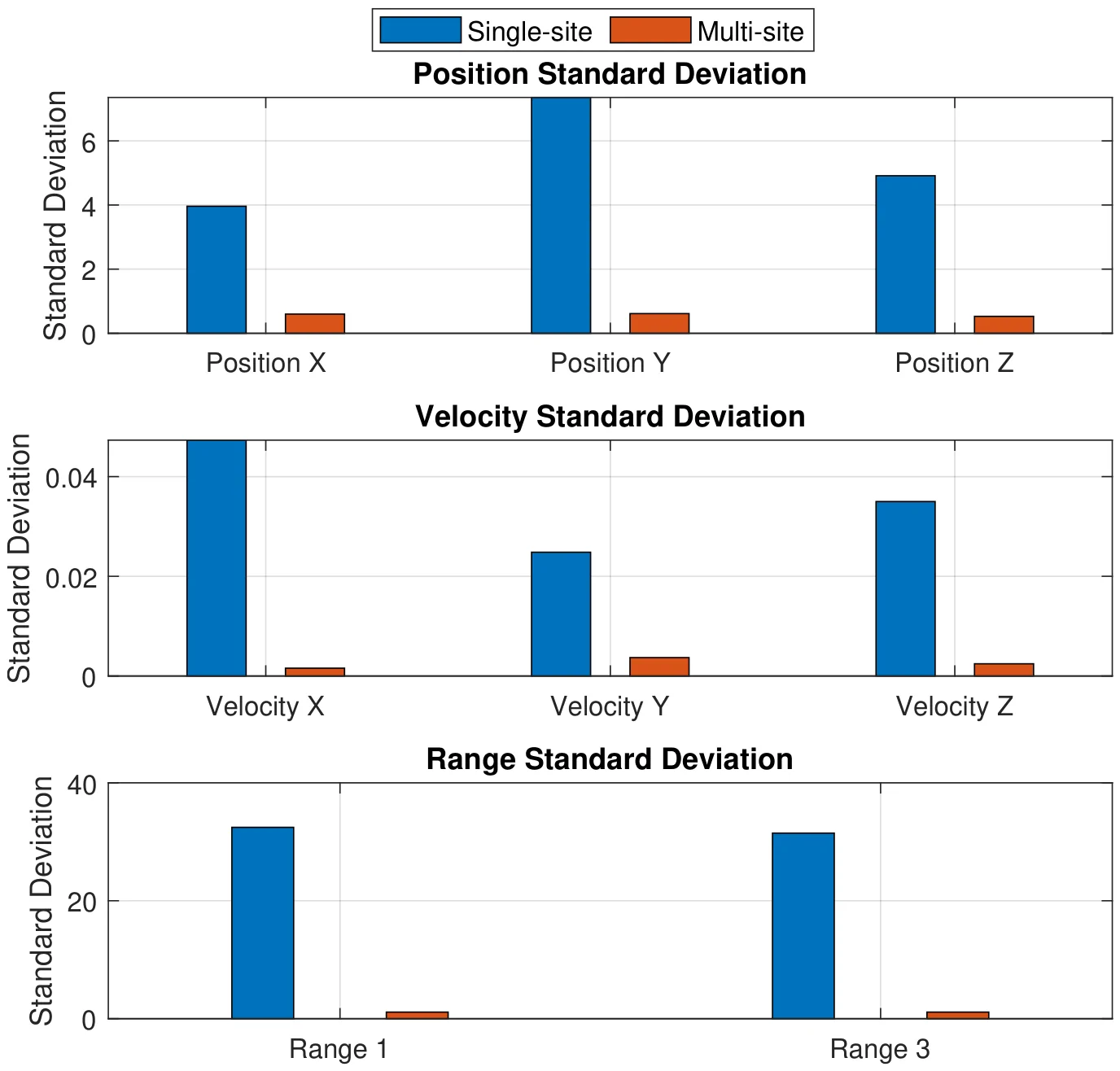
The standard deviation comparison between the single-site scenario (blue) and the ‘simultaneous’ observations from three host satellites in a diagonal-line formation (orange).

Even with some results being more accurate in either three-site or two-site scenarios, it is evident that multi-site observations produce more accurate observations than single-site observations for RSOs in LEO, by far. Even just taking into consideration the ‘simultaneous’ observations from three host satellites in a diagonal-line formation and the single-site observation, this multi-site scenario produced IOD with a decrease in the overall magnitude of error by approximately 88% for position values, 91% for velocity values, and 97% for range values, according to Table 10. This was done using the **Percent RMSE** Equation (3) below, where the absolute value of the numerator was taken to ensure that the negative directions from position and velocity would not affect the overall calculation. To emphasize the difference in error between these two scenarios, Figure 7 displays the RMSE of both scenarios for all result values. For completion, Figure 8 displays the bias, and Figure 9 displays the standard deviation for the same two scenarios.

(1) $$\mathrm{Percent}\;\mathrm{RMSE}=\left[\frac{\left|\text{Single-site}-\text{Multi-site}\right|}{\text{Single-site}}\right]\times 100$$

These single-site versus multi-site results further support the idea in the literature that multi-site observation will produce more accurate IOD results than single-site observation for objects in LEO. These results are extremely important, since the vast majority of unaccounted-for objects are in LEO; thus, the multi-site approach can help with obtaining orbital data and tracking for millions of uncatalogued objects [1].

## 4. Discussion

It is evident, as shown in Table 10, that ‘simultaneous’ three-host-satellite observations in a diagonal-line formation far outperformed the ‘normal’ observations for most other scenarios. This comes as no surprise, since ‘simultaneous’ multi-site observations can obtain more straightforward and accurate range measurements than those of single-site observations.

**Figure 10:**
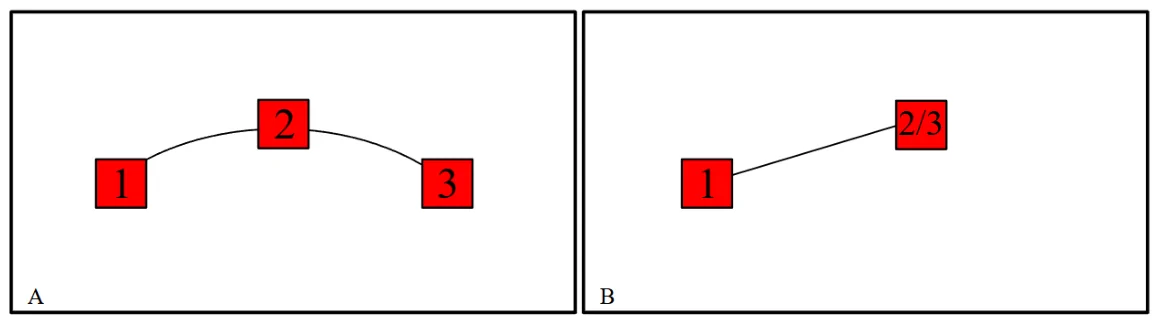
Example of observational arcs. The red boxes represent the RSO being observed. The red boxes with the number ‘1’ represent the first observations. The red box with the number ‘2’ represents the second observation. The red box with the number ‘3’ represents the third observation. The red box with ‘2/3’ represents the second and third observations overlapping. Image (**A**) represents the needed arc of the RSO’s orbit, which is formed from the three observations. Image (**B**) represents the ‘simultaneous’ two host satellites in a diagonal-line formation of the RSO whose data was not suitable for IOD calculations since it does not form an arc.

The ‘simultaneous’ results for two host satellites in a diagonal-line formation, as shown in Table 8, Table 9 and Table 10, are not included in the overall numerical comparison between the different observation scenarios. This is because some RSO data from this scenario could not be successfully processed through Gooding’s algorithm, meaning that a complete set of numerical IOD results could not be obtained for comparison. The convergence-success rate for the gray-line scenario was 90%, while the rest of the scenarios had a convergence-success rate of 100%. Three observations of the RSO need to form a large enough arc of its orbit for the data to be useful in calculating the IOD. As discussed previously, the three observations must be sufficiently separated in time and geometry to form an adequate orbital arc; a too-short arc provides insufficient information to reliably calculate an RSO’s orbit. While inspecting the access reports, it is evident that, for one RSO, its data does not form a large enough RSO arc, thus leading to an improper IOD. Furthermore, this ‘simultaneous’ two-site scenario uses the same input data for two observation times. Examples of this can be seen in Table 2 and Table 4 above. Since the two host satellites observe the RSO simultaneously, two of the three observations provided to the IOD algorithm contain identical input data. Although three observations are supplied to the algorithm, only two are physically distinct observations of the RSO. Thus, the three observations do not provide three separate points along the RSO’s trajectory and, therefore, cannot form an arc. Instead, the observations form only a line, as shown in Figure 10B. Therefore, the inability to properly calculate IOD for this scenario is not the fault of Gooding’s algorithm but is due to the limitations of attempting to calculate IOD from non-independent observations.

Although the numerical results from the ‘simultaneous’ two host satellites in a diagonal-line scenario were excluded from the overall quantitative comparison, they are still retained in this study. The configuration is important for evaluating whether two-site or three-site observations provide a more effective approach for multi-site IOD, which is discussed further in a following paragraph. Removing or modifying this scenario solely because it could not produce IOD solutions would prevent a fair assessment of the conditions under which two-site observations can or cannot provide useful data for IOD calculations. Therefore, the scenario is presented in its original form to clearly demonstrate the limitations of two-site simultaneous observations and to provide a fair basis for comparison with three-site observations.

While the ‘simultaneous’ two host satellites in the diagonal-line scenario were excluded from the overall quantitative comparison, the ‘simultaneous’ two host satellites in the straight-line scenario should be discussed. Although this other scenario had a convergence success rate of 100%, it still contains two identical observation inputs, making the observations look like Figure 10B. An example of this data can also be seen in Table 2. Since the data from this scenario technically cannot be used for IOD calculations, it cannot be quantitatively considered. However, this makes a strong point for our discussion on whether two-site or three-site observations are better for IOD calculations. From the results, it is clear that simultaneous observations are beneficial for LEO RSOs; however, this is true only when the observations were obtained from three hosts. Thus, although the simultaneous two-site scenarios cannot be considered quantitatively overall, their results still remain useful for other analysis regarding the objectives of this research.

Regarding the geometry of the multi-site hosts, the straight-line geometries produced slightly more accurate IOD results than their diagonal-line counterparts. Since the IOD accuracy is heavily influenced by the accuracy of the range estimates, differences in observer geometry may contribute to the observed variation in IOD performance. The observer baseline and orientation affect the relative viewing geometry between the hosts and the RSO, which, in turn, influences the parallax. However, the results do not indicate a consistent advantage for either formation. In particular, one diagonal-line scenario produced substantially more accurate results than the other scenarios tested, demonstrating that diagonal-line configurations can also provide favorable observation geometries. Therefore, the differences in IOD accuracy cannot be attributed solely to the overall straight-line or diagonal-line formation, and the specific LOS geometry between the observers and the RSO should also be considered. Again, it is important to note that the multi-site scenarios tested are idealized observation geometries used to investigate geometric effects, and that formation-flying dynamics and station-keeping are outside the scope of the present study.

To further analyze the specific LOS geometry between observations, Table 12 displays the mean LOS-based geometric dilution of precision (GDOP). The LOS-based GDOP is used as a measure of how the relative direction of the three LOS observations affects the observation geometry. For each RSO, the three LOS unit vectors were arranged in a matrix represented by **H**, and the GDOP was calculated as follows:

(2) $$\mathrm{GDOP}=\sqrt{\mathrm{trace}[{\left(H^{T}H\right)}^{-1}]}$$

The GDOP was calculated separately for each of the 10 RSOs, and the resulting values were then averaged to obtain the mean LOS-based GDOP reported in Table 12. Lower GDOP values indicate a better LOS geometry, while larger values indicate greater geometric dilution and sensitivity to errors in the LOS observations. Regarding the geometry of the multi-site hosts, the results indicate that IOD performance is influenced by the observation geometry; however, the effect cannot be attributed solely to whether the hosts are arranged in a straight-line or diagonal-line formation. The mean LOS-based GDOP values for the ‘normal’ multi-site observations are relatively similar, while the ‘simultaneous’ multi-site observations produce much larger LOS-based GDOP. These results suggest that ‘simultaneous’ LOS geometry has a greater influence on IOD accuracy than the overall observer formation (straight line or diagonal line). Since the ‘simultaneous’ and ‘normal’ cases also differ in observation timing, the current results cannot independently separate the effects of the observation timing and the LOS geometry. Therefore, neither the straight-line nor the diagonal-line geometry can be considered superior based solely on these results, and this would be an important future work topic in the formation-flying research area.

In addition to the constellation geometry, the results for the number of hosts for multi-site observations (either two or three) were taken into consideration. There are two two-site scenarios that were not suitable for comparison, while all three-site scenarios were. This is because three-site observations allow for a larger range of observability, reducing the chance of a too-short-arc problem. Additionally, simultaneous two-site observations suffer the possibility of not forming an arc in observations, as was the case for the ‘simultaneous’ two host satellites in diagonal-line and straight-line formations. Due to this, these two-site scenarios could not be considered quantitatively, while no three-site scenarios suffered from this. Due to the larger range of observability, and considering that the ‘simultaneous’ three host satellites in a diagonal-line formation produced the most accurate IOD results overall, it is safe to conclude that three-site observations are more reliable than two-site observations.

Observation times for multi-site scenarios are also an important topic of comparison. It is evident that ‘simultaneous’ observations are beneficial for observing RSOs in LEO. Simultaneous observations allow for a more accurate and straightforward calculation of range, and greater accuracy in range estimates leads to greater accuracy in IOD calculations. This is supported by the fact that the ‘simultaneous’ observations from three host satellites in a diagonal-line formation produced the most accurate IOD results.

There are a variety of reasons as to why the RMSE of the results is not zero when using simulated data. Firstly, ‘simultaneous’ observations for all observers could only happen when all hosts’ FOVs overlapped, resulting in time constraints. However, it could be seen as beneficial that these near-’simultaneous’ times were not obtained with an exactly zero-second time difference, since absolute ‘simultaneous’ observations are also highly difficult to achieve with real data. Another reason for a non-zero RMSE is due to the IOD algorithm. Although Gooding’s algorithm is one of the most reliable and robust IOD algorithms within the literature, no IOD algorithm can produce flawless results, due to mathematical and logical restraints. One example of this is that Gooding’s algorithm performs better when range estimates are more accurate, even though it iteratively refines these estimates [20]. Nonetheless, Gooding’s IOD is still one of the most robust in the literature.

## 5. Noise Analysis

The simulations presented in this research use the STK truth ephemeris and TLEs to generate the hosts, RSOs, and their corresponding LOS unit vectors [23,24]. This provides a controlled environment for evaluating the effects of host geometry and observation timing on IOD performance. However, actual space-based optical observations contain noise and uncertainties that are not represented by the STK truth data. Some potential error sources include angular measurement nose, optical distortion, attitude determination, pointing errors, host position uncertainties, observational timing errors, and even environmental effects such as target brightness, stray light, and FOV limitations. These factors contribute noise to real data and subsequently propagate through the IOD process.

To investigate some effects of measurement uncertainty on the results presented here, an additional sensitivity analysis was preformed by including Gaussian angular noise in the LOS unit vectors before processing them through Gooding’s algorithm [20,29]. Angular noise levels of 1, 3, 5, and 10 arcseconds were investigated. For each noise level, 1000 independent trails were preformed for each RSO and scenario to account for the variability introduced by random noise. The 0-arcsecond case is included as a reference to the original noise-free simulations analyzed and discussed in Section 3 and Section 4. The angular noise was applied directly to the LOS measurements so that it propagated through the IOD calculation process. It is important to note that this noise analysis perturbs only part of the real measurement chain to create a foundation regarding noise interference with respect to IOD. While still very informative, this noise does not represent a full end-to-end real data performance, as that is beyond the scope of this research but represents an important step for future research. All other simulation parameters and Gooding’s algorithm were kept unchanged [20].

This analysis specifically evaluates the sensitivity of the IOD solution to angular measurement uncertainty and does not represent a complete sensor error model. Rather, it provides a first-order assessment of how noise in the measured target direction affects the resulting IOD solution. The specific effects of other error sources should be discussed in future work.

The noise analysis was performed for two observation configurations: the single-site scenario, and the simultaneous three-site diagonal-line scenario. The single-site scenario was selection because it represents the baseline case for comparison with multi-site observations throughout this study. The simultaneous three-site diagonal-line scenario was selected because it produced the smallest RMSE values of all of the scenarios considered in the noise-free simulations shown in Section 3. As shown previously, this multi-site scenario reduced the overall magnitude of error of the position, velocity, and range errors relative to the single-site scenario, by approximately 88%, 91%, and 97%, respectively. Therefore, comparing these two scenarios under perturbed LOS measurements provides a direct assessment of whether the large improvement observed under idea measurements remains when noise is introduced. Table 13 and Table 14 display the RMSE of the output results from Gooding’s algorithm for varying angular noise.

Table 13 presents the RMSE results for the single-site scenario for increasing levels of noise. The 1- and 3-arcsecond cases produce small changes relative to the noise-free results. At 5 arcseconds, the RMSE increase is larger than for the first two noise levels. Then, at the 10-arcsecond noise interval, the results are quite similar to the noise-free results and show a better RMSE than all other noise levels.

The corresponding results for the simultaneous three-site diagonal-line scenario are presented in Table 14. In the noise-free case, this scenario produces substantially smaller errors than the single-site scenario, which is consistent with the discussion in Section 3 and Section 4. However introducing only 1 arcsecond of angular noise results in a substantial increase in the IOD errors. Increasing the angular noise to 3, 5, and 10 arcseconds produces errors in the order of hundreds or thousands. Unlike the single-site results, the simultaneous three-site diagonal-line scenario shows a very negative response to even the smallest angular noise. This behavior is consistent with the nonlinear IOD problem and the dependence on the quality of the observations.

### Noise Discussion

The noise analysis provides an important extension to the ideal-data results presented in Section 3. It is important to begin this discussion again, noting that this noise analysis perturbs only part of the real measurement chain to create a foundation regarding noise interference with respect to IOD. While still very informative, this noise does not represent a full end-to-end real data performance. Under noise-free conditions, the simultaneous three-site diagonal-line scenario produced substantially smaller IOD RMSE then the single-site scenario. However, the addition of small angular noise produces different results. The simultaneous three-site diagonal-line scenario becomes highly sensitive to the LOS measurement noise, whereas the single-site scenario remains with approximately the same RMSE scale over the tested noise levels. This difference can be related back to the observation geometry discussed earlier in this section. The previous LOS-based GDOP analysis showed that the ‘simultaneous’ multi-site scenarios have substantially larger GDOP values than the corresponding ‘normal’ scenarios. The mean LOS-based GDOP for the simultaneous three-site diagonal-line scenario was 100620, compared to the 95.199 for the normal three-site diagonal-line scenario. These results indicate that the simultaneous observation geometry is highly sensitive to the noise in LOS measurements. However, because the ‘simultaneous’ and ‘normal’ scenarios differ in both observation timing and geometry, the current simulations cannot independently determine whether the increased sensitivity is caused by the timing or the specific LOS geometry.

However, this increase in error should not be interpreted as evidence that multi-site observations are inherently less accurate then single-site observations. It actually demonstrates that the accuracy of multi-site IOD depends strongly on the conditions of the particular observation scenario, meaning that the scenario with the smallest noise-free error is not necessarily the scenario that exhibits the smallest change when noise is introduced.

The present analysis specifically considers random Gaussian angular noise and does not reproduce the full range of error sources encountered by optical space-based sensors. This is because this research is not focused on noise analysis to that extent. Thus, these results should be interpreted as a targeted sensitivity analysis of angular noise. As described in Section 6, other factors that could contribute noise, such as target brightness and pointing errors, should be a focus in future work.

Overall, the noise analysis demonstrates that these simulations provide useful information regarding the potential effect of observation configurations on IOD accuracy, while also demonstrating that the magnitude of the improvement is sensitive to observational noise.

## 6. Conclusions

In the course of this work, IOD accuracy from multi-site observations was studied under a variety of scenarios and compared to that of single-site observations. A main objective of SSA research is improving IOD accuracy to help with RSO congestion and collision predictions; the multi-site IOD results presented here demonstrate the potential for substantial improvements in IOD accuracy under noise-free measurements. With the significant decrease in error calculated in Section 3, the possibility of obtaining the needed orbital accuracy for millions of LEO objects is attainable. However, the additional noise analysis demonstrates that these improvements are sensitive to measurement noise. Additionally, the use of star trackers already in orbit provides multiple space-based observations without the need to launch new satellites and add to the RSO congestion problem. With that, this research supports the use of star trackers not only for RSO observations but also for IOD calculations.

An important aspect of this research is that although simulated data was used, the overall process can be replicated with real data from star trackers. The additional angular noise analysis demonstrated the importance of measurement quality in the IOD process. The analysis emphasizes that even small angular noise errors can heavily influence the overall IOD accuracy, specifically when using the simultaneous three-site diagonal-line scenario. However, this noise analysis was limited to Gaussian angular data and does not represent a full end-to-end real data performance; in real observations, there will be other factors that affect the quality of the data. These constraints could stem from limitations in technology or even overlap inaccuracies for multiple FOVs. With that said, real data can be used through the same process as described in this research; however, the overall quality of the data will highly influence the overall accuracy of the IOD results.

Lastly, this research creates a strong foundation in constellation research for IOD purposes. As described in Section 2.2.2, the scenarios created for multi-site observations were done using a straight-line constellation and a diagonal-line constellation. When comparing the scenarios, the straight-line scenarios performed slightly better than their diagonal-line counterparts. However, in the end, one diagonal-line scenario still significantly outperformed all other scenarios, meaning that the diagonal-line formation cannot be completely cast aside. Additionally, the differences in IOD accuracy cannot be attributed solely to the overall straight-line or diagonal-line formation, and the specific LOS geometry between the observers and the RSO should also be considered. When analyzing the LOS-based GDOP, the results suggest that ‘simultaneous’ LOS geometry has a greater influence on IOD accuracy than the overall observer formation (straight line or diagonal line). Since the ‘simultaneous’ and ‘normal’ cases also differ in observation timing, the current results cannot independently separate the effects of the observation timing and the LOS geometry. Furthermore, the noise analysis further demonstrates that the specific LOS geometry and its sensitivity to noise are important considerations when evaluating multi-site IOD performances. Therefore, neither the straight-line nor the diagonal-line geometry can be considered superior based solely on these results, and this would be an important future work topic in the formation-flying research area. Although these scenarios are idealized observation geometries, they represent a good foundation for future constellation-flying IOD research.

### Future Work

The results from this research serve an important role in supporting the advancements of future SSA projects. The first goal of future work should be testing the scenarios from this work, and possibly other scenarios, using real star-tracker data. RSO detections using star trackers are ideal for this, since these optical observations provide the needed angles-only data required for IOD calculations. While star trackers have been used for RSO detection, this data has not been used for IOD calculations within the literature; hence, this is an important future step. Since there are many star trackers already in orbit, future research could be done by taking the star-tracker images from multiple host satellites and using the optical data to calculate IODs. This would help strengthen these multi-site simulated findings and provide strong support in pushing for future real multi-site observations.

The angular noise analysis presented in this work should be expanded to include a more comprehensive sensor error mode. Future work should investigate additional error and noise sources such as host position uncertainty, attitude determination errors, target brightness variations, pointing errors, and environmental effects such as stray light and FOV obstruction. Varying the magnitude of each of these would allow for a clearer understanding of how each specific noise or uncertainty affects the overall IOD accuracy. Furthermore, parallax-based range estimation should be tested with noisy LOS measurements to determine how this would effect the initial range estimate. This process would be ideal, since it mirrors real optical data closely.

Testing multi-site observations across a broader range of orbits would provide a more comprehensive assessment of their influence in SSA research. Future simulations should consider RSOs located in LEO, MEO, and GEO while maintaining the host(s) in LEO and keeping all other observations and simulation parameters consistent. This approach would isolate the effects of orbital locations, and of the host(s)-to-RSO range, on the IOD performance. For each orbit, single- and multi-site observations should be generated and processed using the same IOD methods, with performance evaluated using position and velocity RMSE and percentage of successful IOD solutions. The relationship between the host(s)–RSO range and IOD accuracy could be quantified by varying the range between the host(s) and RSO to determine how the resulting error changes. The too-short-arc problem could also be investigated using these scenarios. With fixed host(s) and an MEO or GEO RSO configuration, the observation time duration could be increased to evaluate how the observation’s arc size affects the IOD performance. These experiments would extend the current work by quantitatively determining the conditions under which multi-site observations provide a measurable improvement in IOD accuracy with respect to SSA research.

Finally, this research creates a foundation for the starting point of constellation formation with respect to IOD research. Since the results between the two constellations tested within this research cannot rule out either constellation, it would be interesting to test more complex constellations and compare the results. Future work could be done on testing a larger variety of constellation geometry types and determining whether any produce better IOD accuracy from their observations. This research could highlight baseline ranges that produce the most accurate IODs and possibly formulate different ways to combat less ideal baseline ranges as well. Another addition for future work should focus on testing observation timing and geometry separately in order to add onto the foundation that this research provides. While formation flying is beyond the scope of this research, the overall results provide a strong foundation for blending the two research areas together.

## Figures and Tables

**Table 1:** Example of single-site scenario data from access report. This data is for the LEO RSO 15773 and is for single-site observation. ‘CASSIOPE’ is the singular host. The order of observations is dependent on observation time, so it is listed chronologically. The green lines represent ‘normal’ observations [23,24,25].

Host	Observation	Time (H:M:S)	Host Position X (km)	Host Position Y (km)	Host Position Z (km)	RSO Position X (km)	RSO Position Y (km)	RSO Position Z (km)
CASSIOPE	1	08:09:25	−6727.4	−590.23	−2349.7	−2292.1	1802.7	−6559.4
CASSIOPE	2	08:17:24	−4540.3	−967.44	−5164.4	−4556.5	−695.00	−5489.0
CASSIOPE	3	08:24:48	−1423.6	−1087.5	−6547.1	−5673.8	−2871.8	−3291.3

**Table 2:** Example of a multi-site straight line with two-host scenario data from access report. This data is for the LEO RSO 15773 and is for two-site straight-line observations. ‘CASSIOPE_1’ represents the first host, and ‘CASSIOPE_2’ represents the second host. The order of hosts is dependent on observation time, so the hosts are listed chronologically, according to which host saw the RSO first and which host saw it last. The green lines represent ‘normal’ observations, while the yellow lines represent ‘simultaneous’ observations [23,24,25].

Host	Observation	Time (H:M:S)	Host Position X (km)	Host Position Y (km)	Host Position Z (km)	RSO Position X (km)	RSO Position Y (km)	RSO Position Z (km)
CASSIOPE_1	1	08:09:25	−6727.4	−590.23	−2349.7	−2292.1	1802.7	−6559.4
CASSIOPE_2	2	08:17:29	−4570.5	−964.69	−5140.9	−4575.0	−721.22	−5470.0
CASSIOPE_2	3	08:24:54	−1457.5	−1087.6	−6540.8	−5680.5	−2896.2	−3258.0
CASSIOPE_1	1	08:18:24	−4167.7	−998.25	−5433.2	−4770.8	−1008.2	−5251.7
CASSIOPE_2	2	08:18:29	−4199.4	−995.84	−5411.7	−4787.8	−1034.2	−5231.0
CASSIOPE_2	3	08:18:29	−4199.4	−995.84	−5411.7	−4787.8	−1034.2	−5231.0

**Table 3:** Example of multi-site straight line with three-host scenario data from access report. This data is for the LEO RSO 15773 and is for three-site straight-line observations. ‘CASSIOPE_1’ represents the first host, ‘CASSIOPE_2’ represents the second host, and ‘CASSIOPE_3’ represents the third host. The order of hosts is dependent on observation time, so the hosts are listed chronologically, according to which host saw the RSO first and which host saw it last. The green lines represent ‘normal’ observations, while the yellow lines represent ‘simultaneous’ observations [23,24,25].

Host	Observation	Time (H:M:S)	Host Position X (km)	Host Position Y (km)	Host Position Z (km)	RSO Position X (km)	RSO Position Y (km)	RSO Position Z (km)
CASSIOPE_1	1	08:09:25	−6727.4	−590.23	−2349.7	−2292.1	1802.7	−6559.4
CASSIOPE_2	2	08:17:29	−4570.5	−964.69	−5140.9	−4575.0	−721.22	−5470.0
CASSIOPE_3	3	08:25:00	−1491.6	−1087.7	−6534.5	−5687.0	−2920.4	−3224.8
CASSIOPE_1	1	08:18:24	−4167.7	−998.25	−5433.2	−4770.8	−1008.2	−5251.7
CASSIOPE_2	2	08:18:29	−4199.4	−995.84	−5411.7	−4787.8	−1034.2	−5231.0
CASSIOPE_3	3	08:18:33	−4237.4	−992.92	−5385.7	−4801.3	−1054.9	−5214.3

**Table 4:** Example of multi-site diagonal line with two-host scenario data from access report. This data is for the LEO RSO 15773 and is for two-site diagonal-line observations. ‘CASSIOPE_2’ represents the second host, and ‘CASSIOPE_3’ represents the third host. The order of hosts is dependent on observation time, so the hosts are listed chronologically, according to which host saw the RSO first and which host saw it last. The green lines represent ‘normal’ observations, while the yellow lines represent ‘simultaneous’ observations [23,24,25].

Host	Observation	Time (H:M:S)	Host Position X (km)	Host Position Y (km)	Host Position Z (km)	RSO Position X (km)	RSO Position Y (km)	RSO Position Z (km)
CASSIOPE_3	1	08:09:21	−6738.9	−590.80	−2351.0	−2275.3	1817.7	−6561.1
CASSIOPE_2	2	08:17:23	−4542.0	−968.38	−5169.9	−4552.8	−689.75	−5492.7
CASSIOPE_2	3	08:24:49	−1412.5	−1088.3	−6554.4	−5674.2	−2873.1	−3289.5
CASSIOPE_3	1	08:16:21	−4903.5	−933.76	−4879.2	−4312.4	−363.17	−5715.14
CASSIOPE_2	2	08:16:23	−4895.6	−933.44	−4878.8	−4320.5	−373.73	−5708.3
CASSIOPE_2	3	08:16:23	−4895.6	−933.44	−4878.8	−4320.5	−373.73	−5708.3

**Table 5:** Example of multi-site diagonal line with three-host scenario data from access report. This data is for the LEO RSO 15773 and is for three-site diagonal-line observations. ‘CASSIOPE_1’ represents the first host, ‘CASSIOPE_2’ represents the second host, and ‘CASSIOPE_3’ represents the third host. The order of hosts is dependent on observation time, so the hosts are listed chronologically, according to which host saw the RSO first and which host saw it last. The green lines represent ‘normal’ observations, while the yellow lines represent ‘simultaneous’ observations [23,24,25].

Host	Observation	Time (H:M:S)	Host Position X (km)	Host Position Y (km)	Host Position Z (km)	RSO Position X (km)	RSO Position Y (km)	RSO Position Z (km)
CASSIOPE_3	1	08:09:21	−6738.9	−590.80	−2351.00	−2275.3	1817.7	−6561.1
CASSIOPE_2	2	08:17:23	−4542.0	−968.38	−5169.9	−4552.8	−689.75	−5492.7
CASSIOPE_1	3	08:24:48	−1423.6	−1087.5	−6547.1	−5673.8	−2871.8	−3291.3
CASSIOPE_3	1	08:16:21	−4903.5	−933.76	−4879.2	−4312.4	−363.17	−5715.1
CASSIOPE_2	2	08:16:23	−4895.6	−933.44	−4878.8	−4320.5	−373.73	−5708.3
CASSIOPE_1	3	08:16:24	−4893.5	−932.51	−4873.4	−4324.5	−379.01	−5704.9

**Table 6:** Examples of the main inputs for Gooding’s algorithm from LEO single-site observations of RSO 15773 [20].

Inputs	Input Values
Time between first and second observation (s)	479
Time between first and third observation (s)	923
Position vector of host at time 1 (km)	(−6727.4, −590.23, −2349.7)
Position vector of host at time 2 (km)	(−4540.3, −967.44, −5164.4)
Position vector of host at time 3 (km)	(−1423.6, −1087.5, −6547.1)
LOS unit vector of RSO at time 1	(6.7543 × $10^{-1}$, 3.6441 × $10^{-1}$, −6.4107 × $10^{-1}$)
LOS unit vector of RSO at time 2	(−3.8245 × $10^{-2}$, 6.4240 × $10^{-1}$, −7.6540 × $10^{-1}$)
LOS unit vector of RSO at time 3	(−7.5312 × $10^{-1}$, −3.1617 × $10^{-1}$, 5.7691 × $10^{-1}$)
Range estimate at time 1 (km)	6566.5
Range estimate at time 3 (km)	5643.4

**Table 7:** Examples of the outputs from Gooding’s algorithm for LEO single-site observations of RSO 15773 [20].

Outputs	Output Values
Position vector of RSO at time 2 (km)	(−4556.4, −696.98, −5486.6)
Velocity vector of RSO at time 2 (km/s)	(−3.6345, −5.2098, 3.7070)
Range estimate at time 1 (km)	6507.5
Range estimate at time 3 (km)	5590.3

**Table 8:** The bias of output results from Gooding’s algorithm for all nine scenarios for objects in LEO [20,23,24,25,29]. The Position and Velocity vectors are represented by their corresponding XYZ values, while Range 1 represents the range at time 1 and Range 3 represents the range at time 3. Green cells mean that that particular scenario has the best result within that column. Rows that are gray mean that the specific scenario cannot be represented by the data properly.

Simulation Scenario	Position X (km)	Position Y (km)	Position Z (km)	Velocity X (km/s)	Velocity Y (km/s)	Velocity Z (km/s)	Range 1 (km)	Range 3 (km)
Single-site	7.2928 × $10^{-1}$	2.3349	−2.6280	1.5827 × $10^{-2}$	−9.6171 × $10^{-3}$	9.5760 × $10^{-3}$	13.128	9.6881
2-site Straight Line	9.6174 × $10^{-1}$	8.2882 × $10^{-1}$	−1.1906	1.7344 × $10^{-2}$	−8.8601 × $10^{-3}$	8.1718 × $10^{-3}$	10.055	8.8918
Simultaneous 2-site Straight Line	9.3373 × $10^{-1}$	−3.8529 × $10^{-1}$	2.3938	−8.6353 × $10^{-3}$	2.6017 × $10^{-2}$	5.3558 × $10^{-3}$	−6.0563	−6.0934
2-site Diagonal Line	−1.2769 × $10^{-1}$	1.4561	−4.4063	−2.6088 × $10^{-3}$	−3.4387 × $10^{-2}$	5.2354 × $10^{-2}$	50.620	41.995
Simultaneous 2-site Diagonal Line	−1.1024	−1.9672	3.3618	1.3255 × $10^{-2}$	2.3832 × $10^{-3}$	1.1368 × $10^{-2}$	−5.9905	−6.0139
3-site Straight Line	4.0801 × $10^{-1}$	7.9650 × $10^{-1}$	−9.0617 × $10^{-1}$	1.7814 × $10^{-2}$	−7.5184 × $10^{-3}$	4.7846 × $10^{-3}$	7.1773	6.6490
Simultaneous 3-site Straight Line	9.5715	−8.3705	23.080	−1.9171 × $10^{-1}$	3.3431 × $10^{-1}$	−2.9466 × $10^{-1}$	−44.884	−34.077
3-site Diagonal Line	3.7591 × $10^{-1}$	9.6635 × $10^{-1}$	−4.5179	1.9231 × $10^{-3}$	−3.3696 × $10^{-2}$	5.0251 × $10^{-2}$	49.142	39.954
Simultaneous 3-site Diagonal Line	2.9195 × $10^{-1}$	3.4222 × $10^{-1}$	2.9993 × $10^{-2}$	−5.2647 × $10^{-4}$	−1.0459 × $10^{-3}$	1.1198 × $10^{-3}$	4.9897 × $10^{-2}$	4.4948 × $10^{-2}$

**Table 9:** The standard deviations of output results from Gooding’s algorithm for all nine scenarios for objects in LEO [20,23,24,25,29]. The Position and Velocity vectors are represented by their corresponding XYZ values, while Range 1 represents the range at time 1 and Range 3 represents the range at time 3. Green cells mean that that particular scenario has the best result within that column. Rows that are gray mean that the specific scenario cannot be represented by the data properly.

Simulation Scenario	Position X (km)	Position Y (km)	Position Z (km)	Velocity X (km/s)	Velocity Y (km/s)	Velocity Z (km/s)	Range 1 (km)	Range 3 (km)
Single-site	3.9614	7.3540	4.9131	4.7318 × $10^{-2}$	2.4833 × $10^{-2}$	3.4993 × $10^{-2}$	32.454	31.468
2-site Straight Line	2.5241	6.3472	3.3550	4.2867 × $10^{-2}$	2.2564 × $10^{-2}$	3.3765 × $10^{-2}$	30.016	28.461
Simultaneous 2-site Straight Line	3.4684	3.6797	2.8837	2.2027 × $10^{-2}$	3.1574 × $10^{-2}$	2.3654 × $10^{-2}$	3.0662 × $10^{-1}$	3.6179 × $10^{-1}$
2-site Diagonal Line	4.8818	7.6099	3.9321	8.2622 × $10^{-2}$	2.6863 × $10^{-2}$	4.7012 × $10^{-2}$	32.536	30.661
Simultaneous 2-site Diagonal Line	2.6416	3.3106	2.0829	2.3160 × $10^{-2}$	3.0736 × $10^{-2}$	4.4892 × $10^{-2}$	1.5016 × $10^{-1}$	1.5498 × $10^{-1}$
3-site Straight Line	2.5954	6.0460	3.2264	4.8072 × $10^{-2}$	2.4434 × $10^{-2}$	4.0514 × $10^{-2}$	33.829	31.321
Simultaneous 3-site Straight Line	25.727	35.004	48.866	6.6938 × $10^{-1}$	8.8695 × $10^{-1}$	9.6720 × $10^{-1}$	110.47	77.758
3-site Diagonal Line	4.6104	7.6412	3.7578	8.1360 × $10^{-2}$	3.0061 × $10^{-2}$	5.1253 × $10^{-2}$	36.133	33.653
Simultaneous 3-site Diagonal Line	5.9917 × $10^{-1}$	6.1403 × $10^{-1}$	5.2861 × $10^{-1}$	1.5618 × $10^{-3}$	3.6922 × $10^{-3}$	2.4478 × $10^{-3}$	1.1150	1.1111

**Table 10:** The root-mean-square error (RMSE) of output results from Gooding’s algorithm for all nine scenarios for objects in LEO [20,23,24,25,29]. The Position and Velocity vectors are represented by their corresponding XYZ values, while Range 1 represents the range at time 1 and Range 3 represents the range at time 3. Green cells mean that that particular scenario has the best result within that column. Rows that are gray mean that the specific scenario cannot be represented by the data properly.

Simulation Scenario	Position X (km)	Position Y (km)	Position Z (km)	Velocity X (km/s)	Velocity Y (km/s)	Velocity Z (km/s)	Range 1 (km)	Range 3 (km)
Single-site	3.8282	7.3570	5.3508	4.7598 × $10^{-2}$	2.5446 × $10^{-2}$	3.4551 × $10^{-2}$	33.471	31.386
2-site Straight Line	2.5805	6.0782	3.3982	4.4211 × $10^{-2}$	2.3167 × $10^{-2}$	3.3058 × $10^{-2}$	30.199	28.427
Simultaneous 2-site Straight Line	3.4203	3.5121	3.6351	2.2611 × $10^{-2}$	3.9675 × $10^{-2}$	2.3070 × $10^{-2}$	6.0633	6.1030
2-site Diagonal Line	4.6331	7.3648	5.7733	7.8425 × $10^{-2}$	4.2801 × $10^{-2}$	6.8775 × $10^{-2}$	59.288	51.085
Simultaneous 2-site Diagonal Line	2.7236	3.6895	3.8933	2.5544 × $10^{-2}$	2.9076 × $10^{-2}$	4.3824 × $10^{-2}$	5.9922	6.0157
3-site Straight Line	2.4958	5.7908	3.1922	4.8960 × $10^{-2}$	2.4369 × $10^{-2}$	3.8731 × $10^{-2}$	32.886	30.448
Simultaneous 3-site Straight Line	26.216	34.247	51.786	6.6334 × $10^{-1}$	9.0541 × $10^{-1}$	9.6372 × $10^{-1}$	114.00	81.258
3-site Diagonal Line	4.3899	7.3132	5.7550	7.7209 × $10^{-2}$	4.4144 × $10^{-2}$	6.9924 × $10^{-2}$	59.916	51.143
Simultaneous 3-site Diagonal Line	6.3901 × $10^{-1}$	6.7561 × $10^{-1}$	5.0238 × $10^{-1}$	1.5724 × $10^{-3}$	3.6556 × $10^{-3}$	2.5781 × $10^{-3}$	1.059	1.0551

**Table 11:** The RSS errors of position and RSS errors of velocity for all nine scenarios for objects in LEO [20,23,24,25,29]. Green cells mean that that particular scenario has the best result within that column. Rows that are gray mean that the specific scenario cannot be represented by the data properly.

Simulation Scenario	Position (km)	Velocity (km/s)
Single-site	9.8697	6.4084 × $10^{-2}$
2-site Straight Line	7.4263	5.9867 × $10^{-2}$
Simultaneous 2-site Straight Line	6.1031	5.1162 × $10^{-2}$
2-site Diagonal Line	10.442	1.1274 × $10^{-1}$
Simultaneous 2-site Diagonal Line	6.0157	5.8468 × $10^{-2}$
3-site Straight Line	7.0677	6.7015 × $10^{-2}$
Simultaneous 3-site Straight Line	67.394	1.4794
3-site Diagonal Line	10.289	1.1313 × $10^{-1}$
Simultaneous 3-site Diagonal Line	1.0569	4.7416 × $10^{-3}$

**Table 12:** The mean LOS-based GDOP for all multi-site scenarios. The gray row means that the specific scenario cannot be represented by the data properly.

Simulation Scenario	Mean LOS-Based GDOP
2-site Straight Line	80.605
Simultaneous 2-site Straight Line	62.347
2-site Diagonal Line	98.192
Simultaneous 2-site Diagonal Line	179.16
3-site Straight Line	87.175
Simultaneous 3-site Straight Line	19866
3-site Diagonal Line	95.199
Simultaneous 3-site Diagonal Line	100620

**Table 13:** The root-mean-square error (RMSE) of output results from Gooding’s algorithm for the single-site scenario for objects in LEO with varying angular noise [20,23,24,25,29]. The Position and Velocity vectors are represented by their corresponding XYZ values, while Range 1 represents the range at time 1 and Range 3 represents the range at time 3.

Simulation Scenario	Angular Noise (arc-seconds)	Position X (km)	Position Y (km)	Position Z (km)	Velocity X (km/s)	Velocity Y (km/s)	Velocity Z (km/s)	Range 1 (km)	Range 3 (km)
Single-site	0	3.8282	7.3570	5.3508	4.7598 × $10^{-2}$	2.5446 × $10^{-2}$	3.4551 × $10^{-2}$	33.471	31.386
Single-site	1	3.8443	7.3740	5.3766	4.767 × $10^{-2}$	2.5397 × $10^{-2}$	3.4550 × $10^{-2}$	33.468	31.396
Single-site	3	3.8673	7.3754	5.3625	4.8214 × $10^{-2}$	2.5393 × $10^{-2}$	3.4506 × $10^{-2}$	33.545	31.608
Single-site	5	4.0897	7.5002	5.6587	4.6646 × $10^{-2}$	2.4657 × $10^{-2}$	3.4205 × $10^{-2}$	32.932	30.653
Single-site	10	3.8219	7.2051	5.5100	5.1456 × $10^{-2}$	2.6303 × $10^{-2}$	3.8212 × $10^{-2}$	35.295	33.422

**Table 14:** The root-mean-square error (RMSE) of output results from Gooding’s algorithm for the simultaneous 3-site diagonal-line scenario for objects in LEO with varying angular noise [20,23,24,25,29]. The Position and Velocity vectors are represented by their corresponding XYZ values, while Range 1 represents the range at time 1 and Range 3 represents the range at time 3.

Simulation Scenario	Angular Noise (arc-seconds)	Position X (km)	Position Y (km)	Position Z (km)	Velocity X (km/s)	Velocity Y (km/s)	Velocity Z (km/s)	Range 1 (km)	Range 3 (km)
Simultaneous 3-site Diagonal Line	0	6.3901 × $10^{-1}$	6.7561 × $10^{-1}$	5.0238 × $10^{-1}$	1.5724 × $10^{-3}$	3.6556 × $10^{-3}$	2.5781 × $10^{-3}$	1.059	1.0551
Simultaneous 3-site Diagonal Line	1	1690.0	1116.4	1774.9	5.4626	10.563	9.1677	2698.3	2688.0
Simultaneous 3-site Diagonal Line	3	1137.5	1004.7	948.09	4.0805	5.5713	4.9797	1792.4	1786.5
Simultaneous 3-site Diagonal Line	5	1312.3	2090.4	1409.7	4.2728	4.2240	9.0403	2840.6	2844.4
Simultaneous 3-site Diagonal Line	10	1271.0	1422.1	1583.0	4.8651	5.8085	6.3733	2478.4	2478.9

## Data Availability

The STK access report data supporting the results of this study are publicly available on Zenodo at https://doi.org/10.5281/zenodo.20647799 [26]. The Gooding IOD algorithm implementation used in this study is available at [29]. The STK simulation scenario files are available from the corresponding author upon reasonable request, subject to Ansys STK license constraints.

## Abbreviations

The following abbreviations are used in this manuscript:

SSA	Space Situational Awareness
RSO	Resident Space Object
IOD	Initial Orbit Determination
LEO	Low Earth Orbit
MEO	Medium Earth Orbit
GEO	Geostationary Orbit
OD	Orbit Determination
CASSIOPE	Cascade, Smallsat and Ionospheric Polar Explorer
STK	Ansys System Tool Kit
FOV	Field of View
FAI	Fast Auroral Imager
LOS	Line of Sight
RMSE	Root-Mean-Square Error
RSS	Root Sum Square
ECI	Earth-Centered Inertial
GDOP	Geometric Dilution Of Precision
